# 3D Printing of Monolithic Proteinaceous Cantilevers Using Regenerated Silk Fibroin

**DOI:** 10.3390/molecules27072148

**Published:** 2022-03-26

**Authors:** Xuan Mu, Constancio Gonzalez-Obeso, Zhiyu Xia, Jugal Kishore Sahoo, Gang Li, Peggy Cebe, Yu Shrike Zhang, David L. Kaplan

**Affiliations:** 1 Department of Biomedical Engineering, Tufts University, Medford, MA 02155, USA; xuan.mu@tufts.edu (X.M.); constancio.gonzalez_obeso@tufts.edu (C.G.-O.); zhiyux@ymail.com (Z.X.); jugal.sahoo@tufts.edu (J.K.S.); tcligang@suda.edu.cn (G.L.); 2 Division of Engineering in Medicine, Department of Medicine, Brigham and Women’s Hospital, Harvard Medical School, Cambridge, MA 02139, USA; 3 National Engineering Laboratory for Modern Silk, College of Textile and Clothing Engineering, Soochow University, Suzhou 215123, China; 4 Department of Physics and Astronomy, Tufts University, Medford, MA 02155, USA; peggy.cebe@tufts.edu

**Keywords:** 3D printing, self-assembly, printability, structural proteins, salt bath, tyrosine, conformation

## Abstract

Silk fibroin, regenerated from *Bombyx mori*, has shown considerable promise as a printable, aqueous-based ink using a bioinspired salt-bath system in our previous work. Here, we further developed and characterized silk fibroin inks that exhibit concentration-dependent fluorescence spectra at the molecular level. These insights supported extrusion-based 3D printing using concentrated silk fibroin solutions as printing inks. 3D monolithic proteinaceous structures with high aspect ratios were successfully printed using these approaches, including cantilevers only supported at one end. This work provides further insight and broadens the utility of 3D printing with silk fibroin inks for the microfabrication of proteinaceous structures.

## 1. Introduction

3D printing/additive manufacturing of protein-based inks is of particular interest for a broad range of biomedical applications [1,2,3,4,5]. 3D printing offers manufacturing benefits, including digital design and rapid prototyping [6,7,8,9]. Load-bearing proteins, along with polysaccharides and minerals, constitute the primary structural components of living systems, playing essential biomechanical roles [10]. Furthermore, the proteinaceous composition of printing inks may be advantageous in maintaining biocompatibility and biodegradability [2], compared with synthetic polymers. The synergy of 3D printing and protein-based inks is a promising strategy for recapitulating sophisticated human physiology and for developing biomedical devices. Despite substantial progress made in past decades, the mechanical strength and structural integrity of 3D proteinaceous prints remain challenging [11,12].

The challenge of 3D printing with protein-based inks is two-fold. First, conventional 3D printing approaches with synthetic polymer-based inks may not be suitable for proteins. These approaches often involve non-aqueous and non-ambient processing conditions, including high-temperature [13,14], organic solvents [15], and harsh crosslinking reagents such as glutaraldehyde [16,17,18]. These processing conditions may be harmful to the inherent biofunctions of proteins. Other 3D printing approaches involve non-proteinaceous additives, such as polyethylene glycol (PEG) [19,20] and polycaprolactone (PCL) [21,22,23], which may compromise the biological roles of the proteins in the inks.

Second, the biological principles for manufacturing mechanically superior protein tissues/structures remain challenging to recapitulate in vitro. Proteins constitute mechanically superior materials, such as tendons and spider dragline silks [10,24], via the assembly of polypeptide chains at hierarchical length scales [25,26]. The mechanism of the hierarchical molecular assembly should be a valuable source of inspiration for artificial manufacturing approaches with proteins [27,28], yet remains incompletely understood.

In an attempt to address this challenge, we previously developed a 3D printing approach with silk fibroin-constituted inks [27]. Compared with a range of 3D printing approaches with silk fibroin-based inks [29,30,31,32,33], this new approach included a salt bath containing 0.5 M of dipotassium phosphate and 4 M of sodium chloride, which recapitulated native spinning solvent conditions, including dehydration, acidification, and ion gradients. These in vivo solvent conditions regulate the assembly of silk fibroin for silk spinning [34,35,36]. This 3D printing approach is advantageous due to the monolithic protein composition and the improved mechanical performance resulting from the salt-directed hierarchical molecular assembly [27].

Here, we extend our previous work to develop 3D printing approaches with monolithic silk fibroin inks [27]. We characterized the dynamic enrichment process of silk fibroin solutions to produce highly concentrated inks and revealed concentration-dependent fluorescence spectra. The latter provides insights into molecular interactions related to the sol–gel transition during 3D printing. We also printed and characterized 3D silk fibroin prints with complex geometries, high aspect ratios, and cantilevers, providing a significant improvement in printability with protein-based inks [19,20,30,37,38]. Such printability is important to unleash the full potential of 3D printing with protein inks, which can widen the scope of the printable structures and thus enhance utility. In particular, cantilever beams could be incorporated into tissue scaffolds for enhanced porosity and interconnections between cells [15,39,40].

In addition, the 3D designs and the simulations of structural deformation, reported here, could be generally useful for characterizing other inks that can form cantilever-like structures. This work underlies the further development of 3D-printed proteinaceous structures for a broad range of potential biomedical applications.

## 2. Results and Discussion

### 2.1. Preparation of Highly Concentrated Silk Fibroin Solutions as Printing Inks

**Figure 1:**
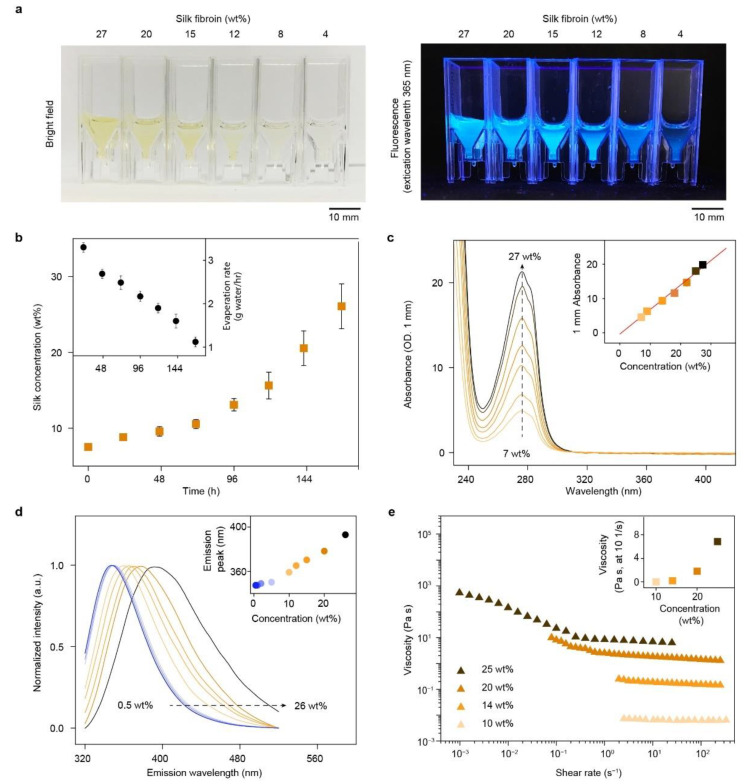
Preparation and characterization of silk fibroin inks. (**a**) Brightfield and autofluorescence images of a series of silk fibroin solutions from 4 to 27 wt%, loaded in cuvettes. Both color and fluorescence intensity increase with increased concentration. (**b**) Enrichment profile of silk fibroin solutions. Inset shows the dynamic change of evaporation rate, defined as the weight of evaporated water per hour. (**c**) Absorption spectra of silk fibroin solutions. Inset shows the linear relationship between 280 nm absorption and concentration. The red line is the linear fitting. (**d**) Fluorescence spectra of silk fibroin solutions. Inset shows emission peak as a function of silk fibroin concentration. (**e**) Viscoelastic profiles of silk fibroin solutions at 10, 14, 20, and 25 wt%. Inset shows that the viscosity at the shear rate of 10 s^−1^ increases exponentially with the increased concentration. The data for 10 wt% regenerated silk fibroin is replotted based on previous results [49]. Reprinted with permission from [49]. Copyright 2014, American Chemistry Society.

Regenerated silk fibroin solutions were prepared from *Bombyx mori* silkworm cocoons including the three steps of degumming, solubilization, and dialysis [41]. The concentration of regenerated silk fibroin solutions was around 8 wt%, a solid content too low to spin/print solid, compact filaments [42]. We thus employed circulating, cooled airflow [42,43] and dialysis cassettes to obtain highly concentrated silk fibroin solutions (over 25 wt%) by water evaporation from solutions to air [27]. A series of silk fibroin solutions were displayed in cuvettes (Figure 1a). The concentration of silk fibroin solution increased from around 7.5 to 26.1 ± 3.0 wt% over 167 h, i.e., around 7 days (Figure 1b). The evaporation rate, defined by the amount of evaporated water per hour, gradually decreased from 3.3 ± 0 to 1.1 ± 0.1 g/h throughout the enrichment process (Figure 1b, inset). The decreased water evaporation was due to the decreased surface area between air and liquid that limits the evaporation, resulting from the decreased volume of silk fibroin solution. In addition, bound water strongly interacts with silk fibroin and is increasingly difficult to remove from the denser solution stages [44,45,46]. This enrichment approach, using low-temperature airflow, can be an alternative to reverse dialysis against PEG (10 wt%, 20 kDa) [47,48]. The polymer solution has a higher osmotic pressure than the silk fibroin solution, thus driving unidirectional water transport from the silk fibroin solution into the polymer solution. Reverse dialysis took around 24 h to achieve 20 wt% silk fibroin and around 3 days to achieve 30 wt% [47,48]. The cooled air approach took around 7 days to achieve 30 wt%, slower than reverse dialysis (Figure 1b). However, the slower enrichment process may be advantageous in reducing premature gelation or aggregation of silk fibroin, improving solution consistency, and minimizing batch-to-batch variations in protein conformation and concentration.

Silk fibroin solutions were darker yellow and had stronger autofluorescence with increased concentration (Figure 1a). The autofluorescence, excited at 365 nm, is consistent with the fluorescence spectra in a previous report [50] and likely results from the increased content of aromatic amino acids of silk fibroin (heavy chain), including tyrosine (5.3 mol%), phenylalanine (0.6 mol%), and tryptophan (0.2 mol%) [51,52]. At 27 wt%, the silk fibroin remained yellowish clear and flowable without turbidity or gelation. The solubility of silk fibroin at high concentrations in aqueous solutions may be attributed to the self-organized micelle structures [53].

### 2.2. Characterization of Silk Fibroin Solutions

A series of silk fibroin solutions was further characterized using ultraviolet-visible (UV-Vis) spectroscopy, fluorescence spectrometry, and rheometry (Figure 1c–e). UV-Vis spectra showed that the absorbance at 280 nm of silk fibroin solution was linearly proportional (R^2^ = 0.99) to concentration from 7 to 27 wt% (Figure 1c). We also used Equation (1) to calculate the percentage extinction coefficient of the regenerated silk fibroin solution (ε1%) that turned out to be 6.8 ± 0.4 (g/100 mL)^−1^ cm^−1^, which may be useful to determine the concentration of regenerated silk fibroin solutions.

The fluorescence spectra of silk fibroin, excited at 300 nm, exhibited notable dependence on concentration (Figure 1d). In particular, with increased concentration from 0.5 to 26 wt%, fluorescence emission exhibited a red shift from 347 to 400 nm and increased full width at half maximum (FWHM) from around 76 to 100 nm (Figure 1d). The concentration-dependent fluorescence spectra were attributed to the tendency of tyrosine residues to self-associate at high concentrations. In particular, the aromatic ring of tyrosine can interact with each other via π–π and π–OH interactions [54,55], which changes energy transfer between tyrosine residues and alters the fluorescence emission. Indeed, the concentration-dependent fluorescence spectra have been found in other fluorophores and attributed to extensive energy transfer [56]. The self-association of tyrosine residues is suggested to play a role in native silk spinning and β-sheet formation by templating the assembly of silk fibroin molecules [57]. These results offer insight that the high concentration of silk fibroin inks provides, in addition to the high solid content, templating effects that facilitate the self-assembly of silk fibroin during 3D printing. Although a higher concentration of silk facilitates 3D printing, it may affect cellular attachment [48]. The decreased attachment with increased silk fibroin concentration is attributed at least in part to increased hydrophobicity of silk on scaffold surfaces [48]. The endothelialization of 3D prints using approximately 30 wt% silk fibroin solution was demonstrated [27]. Thus, concentration–cell attachment relationships require additional investigation.

Rheological measurements for silk fibroin solutions revealed viscoelastic properties relevant to 3D printing (Figure 1e). The viscosity of silk fibroin increased with increased concentrations. At the shear rate of 10 s^−1^, the viscosity of 10, 14, 20, and 25 wt% silk fibroin solutions were 0.01, 0.18, 1.8, and 6.85 Pa∙s, respectively. Silk fibroin solutions below 10 wt% would be more diluted and behave more like water. Viscosity increases exponentially with increased silk fibroin concentration (Figure 1e, inset), consistent with using enriched silk fibroins for electrospinning [49]. The increased viscosity is likely due to increased molecular entanglements with increased concentration. The expedited increase of viscosity after 20 wt% is attributed to a critical entanglement concentration [49]. Furthermore, all four silk fibroin solutions demonstrated shear-thinning behavior, i.e., viscosity decreases with increased shear rate. The shear-thinning behavior is attributed to the elongated molecular chains and the reduced friction between molecules, and is desired for extrusion-based 3D printing, as it lowers the required extruding pressure [2].

### 2.3. Representative 3D Prints with Silk Fibroin Inks

**Figure 2:**
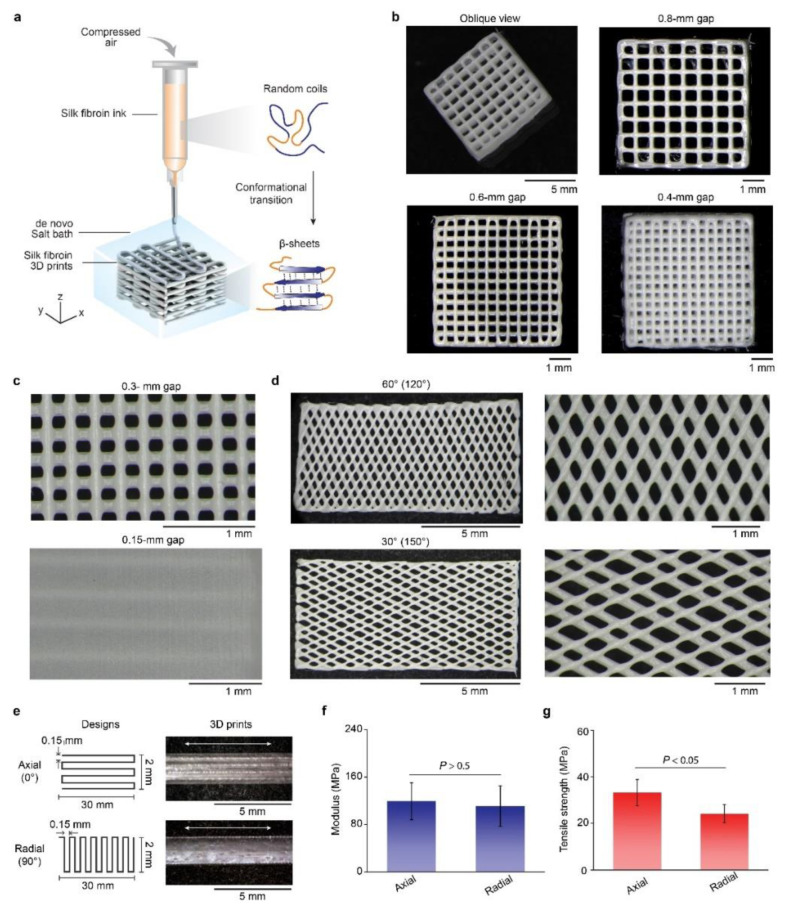
Representative 3D prints with silk fibroin inks. (**a**) Schematic of 3D printing with silk fibroin inks using a salt bath. Silk fibroin transforms from random coils to β-sheet after printing due to the salt bath. (**b**) Optical images of four-layer rectangular lattices with filament gaps from 0.8 to 0.4 mm. (**c**) Optical images of two lattices with filament gaps of 0.3 and 0.15 mm. The latter exhibits closed pores and overlapped filaments, thus becoming a solid film. (**d**) Optical images of two rectangular lattices with distinct filament orientation, including 60° and 30°. (**e**) Design and optical images of two 3D-printed rectangular films (30-mm-long and 2-mm-wide) with 0.15-mm filament gaps and distinct filament orientations, such as axial (0°) and radial (90°). White double arrows indicate the direction of tensile strain. (**f**,**g**) Mechanical performance of the axial and radial films by uniaxial tensile tests, including Young’s modulus and tensile strength.

We used 25 wt% silk fibroin solution as the 3D printing ink, based on prior results (Figure 2a) [27], and printed a 4-layer lattice structure with a footprint of 6 × 6 mm^2^ and filaments of around 100 μm in diameter (Figure 2b). The porosity of the 3D prints was controlled via tuning filament gaps, where gaps of 0.8, 0.6, and 0.4 mm led to the porosities of the 3D prints of 60%, 55%, and 34%, respectively. Furthermore, we used small filament gaps, such as 0.15 mm, to overlap filaments and printed a solid membrane with zero porosity (Figure 2c). The capability to control porosity highlights the promise of 3D silk fibroin prints for tissue engineering, such as for bone regeneration [15,39], as porosity plays a key role in the transport of nutrients and cellular differentiation [40]. In addition, the square shape within the lattice was largely maintained without notable distortions, implying a high printability [58].

The orientation of the silk fibroin filaments in the 3D prints was also controlled, including 0° (axial), 30°, 60°, and 90° (radial) (Figure 2d,e). Filament orientation was associated with mechanical performance. For example, the two rectangle films with axial and radial orientations exhibited Young’s moduli of 119.2 ± 31.0 and 112.3 ± 34.6 MPa, respectively, which are comparable with each other (*p* > 0.5) (Figure 2f). Notably, the tensile strength of the axial films was statistically higher than the radial ones (33.2 ± 5.6 vs. 24.2 ± 3.9 MPa, *p* < 0.05) (Figure 2g). The result of orientation-dependent strength is attributed to the interior interfilament bonding [59], suggesting a means to manipulate the mechanical property of 3D prints. In particular, monolithic silk fibroin prints with controlled filament orientations are promising for the construction of anisotropic tissue scaffolds [60].

**Figure 3:**
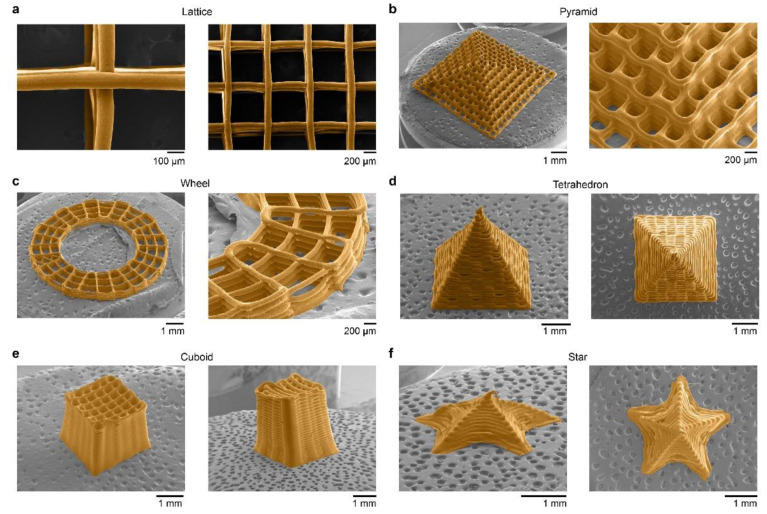
SEM characterizations of representative 3D silk fibroin prints. SEM images of (**a**) the 3D lattices, highlighting single filaments and connections between filaments. (**b**) A 32-layer pyramid-like 3D print. (**c**) An 8-layered wheel-like 3D print. (**d**) A 40-layer tetrahedron-like 3D print. (**e**) A 40-layer cuboid 3D print. (**f**) A single-layer star-like 3D print. Silk fibroin materials are displayed in orange in all SEM images.

We then examined the fine structural features of 3D silk fibroin prints using scanning electronic microscopy (SEM) (Figure 3). 3D-printed lattices exhibited filaments with a round cross-section and well-controlled connections between layers (Figure 3a). Furthermore, other complex 3D silk fibroin prints included a 32-layer pyramid (Figure 3b), an 8-layer wheel (Figure 3c), a 40-layer tetrahedron (Figure 3d), a 40-layer cuboid (Figure 3e), and a 3D star-like shell (Figure 3f). Of note, the star-like shell had no interior supporting structure but only a single shell layer (Video S1), corroborating the printability.

### 2.4. High Aspect Ratio 3D Prints and Cantilevers

**Figure 4:**
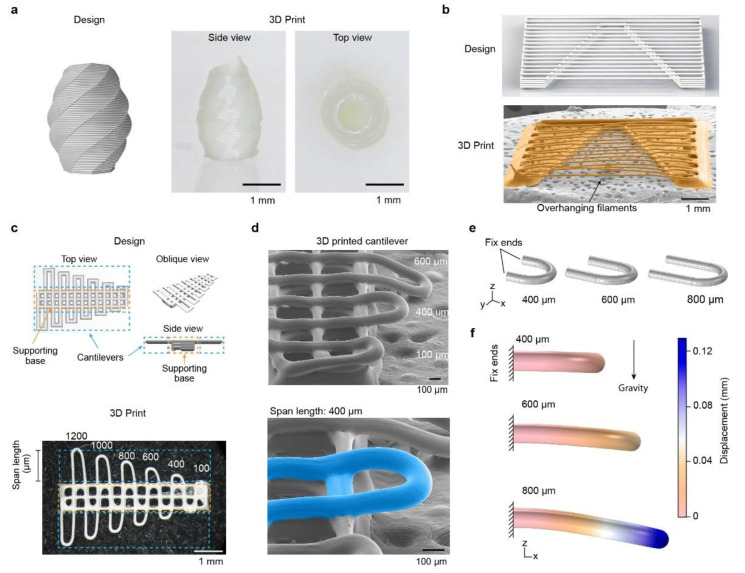
High aspect ratio 3D protein prints and cantilevers. (**a**) A free-standing, hollow, and high-aspect vase-like silk fibroin 3D print. (**b**) A V-shape structure with overhanging filaments for testing printability. The overhanging filaments, ranging from 0.5 to 6 mm in length, remain straight. (**c**) Design and optical image of the cantilever prints, consisting of cantilevers with varying lengths (in cyan dashed boxes) and a 5-layer supporting base (in orange dashed boxes). The span length on one side of the print is labeled in units of microns. (**d**) SEM images of the proteinaceous cantilevers, where the cantilever remains non-sagging for the span length up to 400 μm. (**e**) FEA-based models for the cantilevers with span lengths of 400, 600, and 800 μm. (**f**) FEA simulation results of the cantilever, indicating cantilever deflection due to length.

3D silk fibroin structures were printed with high aspect ratios (Figure 4). One 3D silk fibroin print was a hollow, free-standing, vase-like structure with a slightly convex wall (Figure 4a). The wall thickness and the height of the vase were 0.1 and 2.5 mm, respectively, which led to an aspect ratio of around 25.

A series of overhanging filaments with gradually varied lengths have been used to characterize the printability of concentrated electrolyte-based inks [61] and general bioinks [62]. An overhanging filament may sag or deflect due to gravity, which can be associated with a dynamic sol–gel transition and the strength of the crosslinked ink. A longer filament with minimal sags or deflection angles indicates better print outcomes for the fidelity of the printed structure. On the basis of these methods, we designed and printed a V-shape structure with silk fibroin inks, containing multiple spanning filaments with a constant diameter of around 0.08 mm and lengths varying from 0.8 to 5.6 mm (Figure 4b). In particular, the V-shape structure exhibited no-sagging filaments and had a maximal aspect ratio of around 70. Our previous work exhibited 3D printed silk fibroin filaments with 0.08-mm diameter and 30-mm length, leading to an aspect ratio of over 375 [27]. The high aspect ratio of the 3D silk fibroin prints was superior to polyelectrolyte prints (~20) [61], carbomer-laden hydrogels (~33) [63], and silk fibroin prints based on photo-crosslinking and enzyme-crosslinking (~1) [64,65,66].

In addition to the overhanging filaments (Figure 4b) [61,62], we proposed cantilevers to assess 3D printability (Figure 4c). A cantilever-like structure is challenging to print, as it has to support itself at one end after deposition, in contrast to overhanging filaments supported at both ends. Thus, a cantilever-like structure is a high caliber for 3D printing. We designed a structure composed of a multi-layer supporting base (labeled in orange dashed boxes) and a top layer of continuously printed cantilevers in a U-turn shape with varying span lengths (labeled in cyan dashed boxes) (Figure 4c). The structural design is also useful for assessing the printability of other printing inks that can form cantilever-like structures. We used SEM to evaluate the 3D-printed silk fibroin cantilevers with lengths spanning from 100 to 600 μm (Figure 4d). The silk fibroin cantilevers with up to a 400-μm length span (labeled in cyan) remained straight without sagging. In contrast, the cantilever with a span length of 600 μm exhibited notable deflection.

Finite element analysis (FEA)-based 3D models of proteinaceous cantilevers with span lengths from 400 to 800 μm were developed using the solid mechanics interface in COMSOL (Figure 4e,f) [67]. The 3D models resemble the shape of the cantilever prints. The base layers and the salt batch were omitted in the 3D models. The end boundary of the 3D model, toward the negative x-direction, was fully fixed without displacement (Figure 4e). A gravity load, in the negative z-direction with an acceleration of 9.81 m/s^2^, was applied to the entire 3D model. Experimentally measured and estimated material properties of silk fibroin inks were used in the model, including Young’s modulus (1.9 kPa) and density (1330 kg/m^3^). FEA simulation was used to examine how the cantilever length affected deflection. The simulation results exhibited increased deflection and displacement with increased span lengths of the cantilevers (Figure 4f). In particular, the displacements of the cantilevers with 400-, 600-, and 800-μm lengths were 0.01, 0.05, and 0.13 mm, respectively. The span length-deflection trend in the simulation agreed well with experimental outcomes (Figure 4d). The FEA simulation is a versatile tool to estimate the printability of silk fibroin inks and thus helps design silk fibroin prints with complex structures.

## 3. Materials and Methods

### 3.1. Regeneration and Enrichment of Silk Fibroin Solution

Silk fibroin solution was regenerated from the cocoons of domestic *Bombyx mori* silkworms (Tajima Shoji Co., Yokohama, Japan) according to previously described methods [27,41]. Briefly, 10 g of cocoons were sliced and degummed in 4L of boiling 0.02-M sodium carbonate solution (S7795, Sigma Aldrich, St. Louis, MO, USA) for 30 min. The degummed silk cocoons were dried overnight and solubilized in 9.3-M of lithium bromide (Sigma Aldrich, St. Louis, MO, USA) at 60 °C for 4 h, followed by dialysis (Molecular weight cut-off, MWCO, 3500) against 5-L deionized (DI) water with 6 changes over 3 days. The silk fibroin solution was then centrifuged twice at 9000 rpm, 4 °C for 20 min and filtered through sterile cell strainers (70-µm pore size, Fisherbrand™, Fisher Scientific, Waltham, MA, USA) to remove any insoluble particulates. The degumming and processing conditions gave rise to around 100 kDa of molecular weight of the regenerated silk fibroin, according to previous results [68]. The regenerated silk fibroin solution looked yellowish–clear and remained stable at 4 °C for at least 4 weeks. The silk fibroin solution with the concentration of 7.5 wt% was enriched by air-drying in Slide-a-Lyzer dialysis cassettes (MWCO 3500 Da, Thermo Fisher Scientific) in a refrigerator for about 7 days. We assumed that the refrigerator provides a circulating, cooled (4 °C) airflow and a consistent humidity in the course of the enrichment. The enriched solution remained fluid yet highly viscous, which can be removed from the cassette by scooping or pouring. The concentration of silk fibroin solution was determined by drying at 60 °C overnight using the ratio of the solution weight to the dry weight.

### 3.2. Fluorescence Spectroscopy

Silk fibroin solutions (around 500 µL) were pipetted into a 10-mm-pathlength disposable cuvette (UVette, Eppendorf). Fluorescence spectra of the silk fibroin solution were obtained using an F4500 spectrofluorometer (Hitachi, Schaumburg, IL, USA). The scan rate was 60 nm/min. The excitation and emission slits were 5 and 10 nm, respectively. The voltage of the photomultiplier tube detector was 700 V. The excitation wavelength was 300 nm. The emission from 320 to 500 nm wavelength was collected.

### 3.3. UV-Vis Absorbance

The UV-Vis spectra of silk fibroin solutions from 200 to 800 nm were acquired using a Nanodrop 2000 UV-Vis spectrophotometer (Thermo Fisher Scientific, Wilmington, DE, USA). Each sample was measured at least three times. A wavelength of 340 nm was used for a bichromatic normalization. DI water to dilute the silk fibroin solutions was used as a blank sample. The percentage extinction coefficient of regenerated silk fibroin solutions (ε1%, (g/100 mL)^−1^ cm^−1^) is calculated by using the following equation:

(1) ε1%=A/bc

where *A* is the absorbance at 280 nm, *b* is the path length (cm), and *c* is the percentage concentration (wt%).

### 3.4. Rheology

Steady shear sweeps of silk fibroin solutions with concentrations of 14 wt%, 20 wt%, and 25 wt% were performed on an ARES-LS2 (TA Instruments, New Castle, DE, USA) using a pair of 25-mm parallel stainless steel plates at room temperature. The range of the viscosity-shear rate curve was set according to the limit of torque force and sample stability.

### 3.5. 3D Printing

The silk fibroin solution was printed using an extrusion printer (Inkredible, Cellink, Sweden) and Repetier-Host software, according to previous methods [27]. Briefly, the silk fibroin solution as the printing ink was loaded in a 3-mL syringe and extruded through a 33-gauge chamfered dispensing nozzle by compressed air. The ink was printed on a glass slide that was immersed in an aqueous salt bath (4-M sodium chloride and 0.5-M dipotassium phosphate). The printing path was controlled by manually written G-code commands. The printing speed (moving speed of printing head) and printing pressure were 1 mm s^−1^ and 210 kPa, respectively, unless stated otherwise.

### 3.6. Morphology

3D silk fibroin prints were treated with a series of ethanol solutions, dried in a critical-point dryer (CPD 300, Leica, Germany), and coated with a 5–10-nm-thick Pt/Pd (80:20) (EMS 150 S sputter coater, Quorum, UK), followed by scanning electronic microscopy (SEM) imaging (Ultra 55, Carl Zeiss AG, Germany) at an acceleration voltage of 5 kV.

### 3.7. Tensile Tests

Tensile tests of 3D-printed silk fibroin films were performed using a dynamic mechanical analyzer (RSA3, TA Instruments, New Castle, DE, USA) equipped with a 35-N loading cell at room temperature. The tensile strain rate was 10 mm/min. Young’s modulus was calculated between strains of 0.05 and 0.1 mm/mm. The dimensions of the samples were measured by light microscopy before testing. The samples were immersed in 1 × phosphate-buffered saline (PBS) and remained wet during testing.

### 3.8. Computational Simulation

COMSOL Multiphysics 5.4 (COMSOL, Inc., Burlington, MA, USA) was used to develop an FEA-based model of the proteinaceous cantilever-like structures, according to the actual size. We estimated Young’s modulus (*E*) of the models from the shear modulus (*G*) of silk fibroin inks by assuming *E* = 3*G* [69]. The density of the model was assumed to be the difference between silk fibroin inks and the salt bath.

### 3.9. Statistical Analysis

Microsoft Excel software was used for all statistical analyses. Unless otherwise described in the text, all experiments were repeated as n = 3 independent samples or measurements, and the experimental data are expressed as means ± standard deviation (s.d.). The statistical comparison among two groups was performed using the single-factor ANOVA method. *p*-value larger than 0.1 was recognized as not statistically significant.

## 4. Conclusions

In summary, monolithic silk fibroin solutions, as 3D printing inks, demonstrates useful and unique printability when compared to other protein materials and some synthetic polymers. The characterization of the silk fibroin inks revealed concentration-dependent molecular mechanisms, such as tyrosine interactions, that may play a role in the sol-gel transition of silk fibroin and facilitate 3D printing. Given the inherent biological relevance of protein materials, silk fibroin inks are a viable alternative to synthetic polymers in additive manufacturing for biomedical devices and tissue scaffolds.

## Data Availability

Data are contained within the article.

## Supplementary Materials

The following supporting information can be downloaded at: https://www.mdpi.com/article/10.3390/molecules27072148/s1, Video S1: 3D printing of single-layer star.

## Sample Availability

3D printing designs are available from the authors.
